# Linguistic findings in persons with schizophrenia—a review of the current literature

**DOI:** 10.3389/fpsyg.2023.1287706

**Published:** 2023-11-21

**Authors:** Felicitas Ehlen, Christiane Montag, Karolina Leopold, Andreas Heinz

**Affiliations:** ^1^Department of Neurology, Motor and Cognition Group, Charité – Universitätsmedizin Berlin, Corporate Member of Freie Universität Berlin and Humboldt-Universität zu Berlin, Berlin, Germany; ^2^Vivantes Klinikum am Urban und Vivantes Klinikum im Friedrichshain, Kliniken für Psychiatrie, Psychotherapie und Psychosomatik, Akademische Lehrkrankenhäuser Charité - Universitätsmedizin Berlin, Berlin, Germany; ^3^Department of Psychiatry and Psychotherapy, Campus Charité Mitte (Psychiatric University Clinic at St. Hedwig Hospital, Große Hamburger Berlin) – Universitätsmedizin Berlin, Corporate Member of Freie Universität Berlin, Humboldt-Universität zu Berlin, and Berlin Institute of Health, Berlin, Germany; ^4^Klinik und Poliklinik für Psychiatrie und Psychotherapie, Universitätsklinikum Carl Gustav Carus, Dresden, Germany; ^5^Department of Psychiatry and Neurosciences, Charité – Universitätsmedizin Berlin, Corporate Member of Freie Universität Berlin, Humboldt-Universität zu Berlin, and Berlin Institute of Health, Berlin, Germany

**Keywords:** formal thought disorder, schizophrenia, psychosis, differential diagnostics, early detection, linguistic categories, language, natural language processing

## Abstract

**Introduction:**

Alterations of verbalized thought occur frequently in psychotic disorders. We characterize linguistic findings in individuals with schizophrenia based on the current literature, including findings relevant for differential and early diagnosis.

**Methods:**

Review of literature published via PubMed search between January 2010 and May 2022.

**Results:**

A total of 143 articles were included. In persons with schizophrenia, language-related alterations can occur at all linguistic levels. Differentiating from findings in persons with affective disorders, typical symptoms in those with schizophrenia mainly include so-called “poverty of speech,” reduced word and sentence production, impaired processing of complex syntax, pragmatic language deficits as well as reduced semantic verbal fluency. At the at-risk state, “poverty of content,” pragmatic difficulties and reduced verbal fluency could be of predictive value.

**Discussion:**

The current results support multilevel alterations of the language system in persons with schizophrenia. Creative expressions of psychotic experiences are frequently found but are not in the focus of this review. Clinical examinations of linguistic alterations can support differential diagnostics and early detection. Computational methods (Natural Language Processing) may improve the precision of corresponding diagnostics. The relations between language-related and other symptoms can improve diagnostics.

## 1 Introduction

Language comprehension and production are among the most complex brain functions in humans and seem to follow fundamental governing principles (Sportiche et al., 2014). By communicating, language provides a link between the externally perceivable and internal thought processes. In psychiatry, observations of altered language are traditionally taken as indications of underlying alterations in thought. In particular, schizophrenia can be accompanied by severe alterations of language functions. Idiosyncratic psychotic experiences can also lead to unusual verbal expressions that are often highly creative (Heinz, 2023). However, traditional accounts understand linguistic alterations in persons with schizophrenia as deficits directly caused by the psychotic disorder, and our review will also focus on this approach, while recognizing the need to also address the creative use of language in future studies.

With respect to a deficit-oriented account of linguistic alterations, explanatory models of verbal expression as a manifestation of an impaired thought process go back to, among others, the theoretical accounts of Kraepelin (1896) and Bleuler (1911a). According to Bleuler, the coincidence of disturbed regular associations and random combinations of only superficially related ideas would lead to “disjointed” (“zerfahren”), “bizarre,” “incorrect,” and “abrupt thinking” with “loss of goal” (Bleuler, 1911b). Further, an (especially subjective) increase of associatively altered thoughts (“Gedankendrängen”) was contrasted to an (objective and subjective) “blocking of thoughts” (“Sperrung”; Kraepelin, 1896), which—together with “poverty of ideas”—Bleuler regarded as fundamental for the symptomatology (Bleuler, 1911c). In addition to these association disorders, he classified, e.g., a “wrong” choice of words, a disturbed formation of new and “wrong” words as well as grammar violations as genuine linguistic disorders, which in the most severe cases could lead to completely incomprehensible “word salad” (Bleuler, 1911c). These symptoms—which could also be found in a mild form in healthy people—could occur only episodically in people with schizophrenia, sometimes with mild symptoms, sometimes with clear disturbances, up to high-grade association disturbances, which would ultimately result in incoherence (“Zerfahrenheit”) (Bleuler, 1911b). Jaspers (1946) distinguished incoherence from an often witty or creative flight of ideas. Yet, seemingly incoherent or only loosely connected utterances are found in modern poetry or may result from anxiety and differences in social status, power and education (Wübben, 2012; Heinz, 2023). Accordingly, Schneider (1950) and Muleh and Carpenter (1974) and in this tradition ICD-10 (World Health Organization, 1993) focuses on language-related symptoms reported by the patients themselves (such as thought insertion or complex acoustic hallucinations) rather than impressions of coherence by psychiatrists—a critical approach that will be addressed in the discussion of this review.

A systematization of language-related findings as “positive” (i.e., pressure of speech, tangentiality, derailment, incoherence and illogicality) and “negative” (i.e., poverty of speech and poverty of content of speech) symptoms using the *Scale for the Assessment of Thought, Language, and Communication* (*TLC*) (Andreasen, 1979a; see Box 1) reflects evolutionary speculations of (Jackson 1884) (for a criticism see Heinz, 1998). It promotes a characterization of subtypes and severity levels across diagnostic groups (Andreasen, 1979b; Andreasen and Grove, 1986).

**BOX 1 Selection of language-specific clinical scales.**

Scale for the assessment of thought, language, and communication (TLC) (Andreasen, 1979a; Andreasen, 1986)

- Standard interview of about 45 min
- 18 subtypes of thought disorders
- Severity per item from 0 to 3 or from 0 to 4
- Designed to examine FTD in schizophrenia, mania, schizoaffective disorder, depression, and in healthy individuals
- Three symptom domains delineable (Fluent disorganization, Emptiness, Linguistic control) (Andreasen and Grove, 1986)
- Includes definitions and instructions for scoring severity

Thought and Language Disorder scale (TALD) (Kircher et al., 2014)

- 50 min open and semi-structured interview
- 30item scale
- Severity per item 04
- Based on all symptoms of formal thought disorders described in the literature since the early twentieth century
- Designed to assess FTD in schizophrenia, mania, depression and in healthy individuals
- Four delineable symptom domains 1: Objective positive, 2: Subjective negative, 3: Objective negative, 4: Subjective positive

Thought and Language Index (TLI) (Liddle et al., 2002)

- Eight 1-min speech samples
- Eight assessment categories: 2 × Impoverishment (Poverty of speech, Weakening of goal), 4 × Disorganization (Looseness, Peculiar word, Peculiar sentence, Peculiar logic), Distractibility, Perseveration
- Severity per item 0.25–1
- Specific for the assessment of FTD in persons with schizophrenia

Communication Disturbances Index (CDI) (Docherty et al., 1996)

- Semi-structured interview
- Six types of communication errors (based on the concept of the unclear reference): 1. Vague references, 2. Confused references, 3. Missing information references, 4. Ambiguous word meanings, 5. Wrong word references, 6. Structural unclarities
- Counts all cases in which the meaning of a word or phrase is unclear, any incorrect word usage, and any incorrect grammatical structure that affects intelligibility.
- Subtly unclear meanings and words or phrases whose definition is ambiguous or unclear in context are also counted.
- The total score is given as the sum of all six types of errors per 100 words.

**Figure 1:**
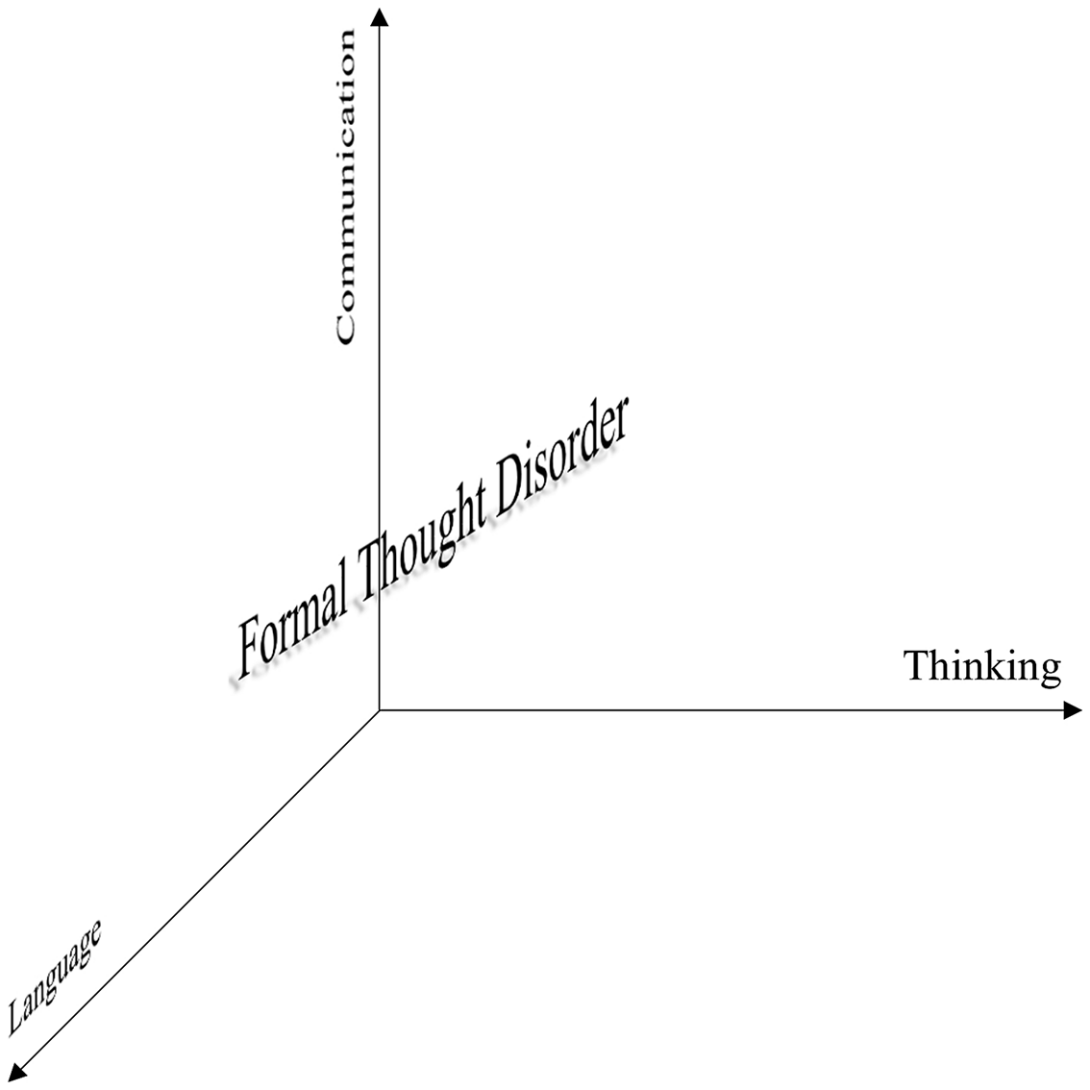
Illustration of formal thought disorder conceptualized by Hart and Lewine (2017) as a “multidimensional construct” with dimensions of thought, language, and communication.

Conceptually, clinically describable formal thought disorders (FTD) can be understood as a “multidimensional construct” encompassing alterations in thinking (including organization, control, processing, and expression), language, and communication (Hart and Lewine, 2017) (for an illustration see Figure 1).

The language-related manifestation of FTD motivated descriptions on the basis of linguistic categories. These generally comprise phonology, morphology, syntax, lexical semantics, and pragmatics with their respective sub-processes (e.g., Ahlsén, 2006). From this perspective, individuals with schizophrenia have been found to exhibit primarily altered pragmatics, with, e.g., impaired cohesion and coherence, as well as altered lexical access, formation of neologisms, stilted speech, and association chaining (Covington et al., 2005). In contrast, morphology and basic syntax have been described as largely unaffected (Covington et al., 2005), as has phonology (Covington et al., 2005, *cf.* Parola et al., 2023, but *cf.* de Boer et al., 2023; Voppel et al., 2023).

Reflecting the conceptual levels of thought production and verbalized expression, “disorganized speech” (American Psychiatric Association, 2013) and “thought disorder” (WHO, 2019) entered the current diagnostic manuals as leading symptoms of schizophrenia and are recorded as positive and negative FTD in common clinical scales—especially in the *Scale for the Assessment of Negative Symptoms* (*SANS*) (Andreasen, 1983) and the *Scale for the Assessment of Positive Symptoms* (*SAPS*) (Andreasen, 1984) as well as the *Positive and Negative Syndrome Scale* (*PANSS*) (Kay et al., 1987) and the *Structured Interview for Psychosis-risk Syndromes* (*SIPS*) (McGlashan et al., 2001). In addition, a number of language-specific scales have been developed to assess FTD in detail (for a selection see Box 1).

### 1.1 Aim of the review study and search strategy

While we acknowledge and respect the creativity in linguistic expressions of psychotic experiences, this review focuses on linguistic findings considered as pathological in persons with schizophrenia to approach the following questions:

- Which linguistic phenomena are found in persons with schizophrenia?
- Which findings have predictive utility?
- Is it possible to distinguish more disease-specific from transdiagnostic phenomena?

For this purpose, recent investigations of FTD on the basis of clinical scales as well as investigations on the linguistic levels of lexico-semantics, syntax, and pragmatics will be presented. In addition, an overview of the currently rapidly growing findings from natural language processing (NLP) will be given followed by studies focusing on early detection and comparative studies.

A literature search was performed via PubMed [PubMed (nih.gov)] with limitation of the search period from 2010 to May 2022 using the following search terms:

- (language) OR (speech) AND (schizophrenia); this yielded 2,724 results
- (speech) OR (language) AND (differential diagnosis) AND (schizophrenia) (all fields); this yielded 71 results
- (speech) OR (language) AND (differential diagnosis) AND (psychosis) (all fields); this yielded 68 results
- (language) AND (differential diagnosis) AND (psychiatry) (all fields); this yielded 216 results; of these, those related to schizophrenia/psychotic disorders were manually selected
- (“formal thought disorder”) AND (“differential diagnosis”) (all fields): this yielded 2 results
- Inclusion criteria were:
- Studies focusing on clinical speech or language assessment (including production or processing) in adults diagnosed with schizophrenia, psychotic disorders, or schizophrenia spectrum disorder
- Studies focusing on clinical speech or language assessment in persons at high risk of schizophrenia/psychosis
- Studies focusing on speech or language assessment in adults diagnosed with schizophrenia compared with persons diagnosed with either bipolar disorder and/or schizoaffective disorders, and/or depressive disorder
- Primary Research
- Reviews and Meta-Analyses that reflected the most current research were included
- Article written in English or German
- Exclusion Criteria were:
- Case Reports
- Studies focusing on children/adolescents diagnosed with schizophrenia/psychosis
- Studies with main focus on neuroimaging or genetics
- Studies exclusively on motor speech function/prosody
- Studies exclusively on “inner speech” or acoustic hallucinations
- Studies exclusively on speech therapy
- Studies focusing on the specifics of a particular language (including sign language) or on bilingualism
- Studies on body language
- Studies of texts of affected persons published on the internet (e.g., on “social media”)
- After deleting duplicates, this resulted in a total of 143 included studies (including 11 reviews and 5 meta-analyses; all articles were written in English).
- Regarding terminology, the term “psychosis” has been adopted in this review when used by the authors of the reported studies without more specificity.

## 2 Current clinical findings

### 2.1 Findings based on clinical scales

Studies from the current literature search reported prevalences of 55% FTD in the early stages of psychotic disorders (Oeztuerk et al., 2022) or 19% FTD at first episode of schizophrenia (Roche et al., 2015), and overall 27% clinically impairing FTD in schizophrenia (Roche et al., 2015). Conceptually, the presence of FTD already in early phases of psychotic illness has been suggested to be a “marker of ‘psychosis proneness’,” whereas the acute manifestation of FTD may represent a “breakdown” in language structure (Roche et al., 2015).

The discriminability between positive FTD (especially tangentiality, derailment, incoherence, pressure of speech, and illogical thinking) and negative FTD was replicated in current studies using both language-specific scales (*TLC* in Comparelli et al., 2020, *TALD* in Kircher et al., 2014) and the *SAPS/SANS* (Roche et al., 2016; Ucok et al., 2021). This repeatedly showed a predominance of positive FTD in the early phases of psychotic illness, which tend to decline over time, whereas negative FTD often persist in the further course of the disease (Roche et al., 2016; Comparelli et al., 2020; Ucok et al., 2021).

Whereas the prevalence of positive FTD has been associated with worse clinical outcome [in terms of overall symptomatology (Wilcox et al., 2012; Little et al., 2019), hospitalization (Roche et al., 2016), and occupational functioning (Muralidharan et al., 2018)], contradictory results suggested both independence from cognitive functions (Comparelli et al., 2020; Fuentes-Claramonte et al., 2021) versus associations with specific cognitive impairments [involving goal maintenance (Becker et al., 2012), executive functions (Little et al., 2019; Oeztuerk et al., 2022), problem solving (Little et al., 2019), and attention (Oeztuerk et al., 2022)]. Clinically, positive FTD tended to intensify when talking about negatively connoted content (so-called affective reactivity of language; Docherty et al., 1994; Minor et al., 2016).

Negative FTD, on the other hand, has been associated not only with decreased quality of life (Bowie et al., 2011; Tan et al., 2014), but also more unequivocally with cognitive impairments [involving verbal working memory (Becker et al., 2012; Nagels et al., 2016; Ucok et al., 2021), problem-solving (Comparelli et al., 2020), and attention (Nagels et al., 2016; Ucok et al., 2021)] and illogicality (as assessed by the *TLC*) (Comparelli et al., 2020). Moreover, the global severity of FTD seemed related to impairments in social functioning (Comparelli et al., 2020; Oeztuerk et al., 2021; meta-analysis, see Marggraf et al., 2020), where negative FTD could determine the observable behavior and positive FTD the performance (“appropriateness”) in social interactions (as assessed by the *Specific Level of Function Scale*; Schneider and Struening, 1983; Bowie et al., 2011). Lastly, “peculiar use of words” and “peculiar logic” in the *Thought and Language Index* (*TLI*) (Liddle et al., 2002, see Box 1) that contributed to the distinction between individuals with and without first psychotic episode (Ayer et al., 2016) or with and without clinical remission (Yalınçetin et al., 2016) may have independent diagnostic value.

*In summary* assessments by clinical scales indicate a preponderance of positive FTD in the early phase of schizophrenia, while negative FTD tend to prevail in the further course, leading to impairments in about a third of the patients. While studies found both to be associated with further disease-related impairment in cognitive and social functioning, positive FTD have among others been related to greater clinical burden and negative FTD to lower quality of life.

### 2.2 Findings from linguistic analyses

#### 2.2.1 Lexico-semantics

Analyses at the lexico-semantic level usually assume a network model of semantic memory, in which the strength of connections between individual semantic “entries” (memory items) is determined by their shared use or conceptual similarity (Collins and Loftus, 1975; Levelt, 1999; Rofes et al., 2019). According to this, word retrieval at the initial level of abstract conceptualization (e.g., Binder and Desai, 2011; Kiefer and Pulvermüller, 2012) and the subsequent level of lexico-semantic access (e.g., Pulvermüller, 1999) leads to automatic spreading activation within the network, so that co-activated associated entries will be accessed more quickly (so-called priming) and must in turn be inhibited during the final selection (Collins and Loftus, 1975; Dell, 1986).

##### 2.2.1.1 Lexico-semantics (processing)

Against this background, lexical decision tasks, which require participants to indicate whether a target stimulus presented after a so-called prime word is a real word or not (e.g., *house* vs. *frouse*), allow quantification of word relatedness effects. Here the presentation of target words related to the prime leads to shorter reaction times than the presentation of unrelated words (Meyer and Schvaneveldt, 1971). This is expressed by the priming effect (Neely, 1991), where hyperpriming refers to an excessive difference between reaction time for unrelated compared to related words and hypopriming to a **r**educed priming effect.

In simultaneous EEG recordings of event-related potentials (ERPs), an additional reduction in the amplitude of the N400 component is observed when related words are presented, the so-called N400 effect (Bentin et al., 1985). In this context, the N400—a negativity with peak amplitude at approximately 400 ms after stimulus onset—most likely reflects the neuronal activity resulting from a temporary “binding” between the stimulus and the pre-activated semantic network (Kutas and Federmeier, 2011).

Early influential findings indicated hyperpriming in persons with schizophrenia with FTD (as assessed by “conceptual disorganization” in the *Brief Psychiatric Rating Scale;* Overall and Gorham, 1962) both when semantic stimuli were directly and only indirectly [by an implicit mutual association, e.g., “lemon-(sour)-sweet”] related (Spitzer, 1993; Spitzer et al., 1993). The latter was evoked specifically at short interstimulus intervals (the so-called stimulus onset asynchrony; SOA), which are thought to provoke automatic semantic activation processes rather than controlled activation (which is provoked by longer intervals). This was suggested as “indicator of associative network dysfunction” (Spitzer et al., 1993) with an “overinclusive” network leading not only to a more readily activation of close but also of more distant associations (Spitzer, 1993).

Regarding more recent findings, a current review reported heterogeneous results (Almeida and Radanovic, 2021): Hyperpriming, described by the majority of studies and associated with positive FTD (e.g., Safadi et al., 2013; Kuperberg et al., 2019), was repeatedly reported (Almeida and Radanovic, 2021), whereas other studies found persistent (i.e., beyond the acute phase of illness; Besche-Richard et al., 2014) hypopriming (e.g., Niznikiewicz et al., 2010; Sass et al., 2014; Tan et al., 2015). Furthermore, the typicality effect (i.e., faster response to typical than to atypical words) was found to be reduced during the initial episode of schizophrenia spectrum disorder (but not during stabilization) and correlated with negative symptoms (Hui et al., 2012).

Also with regard to N400 findings, review papers pointed to incongruent study results (Kuperberg et al., 2010; Mohammad and DeLisi, 2013) without a clear correlation between specific N400 patterns and FTD measures (Almeida and Radanovic, 2021). Here, persons with schizophrenia predominantly showed a reduced N400 effect (e.g., Laurent et al., 2010; Mathalon et al., 2010; Niznikiewicz et al., 2010; Kiang et al., 2014; meta-analysis see Wang et al., 2011; review see Almeida and Radanovic, 2021), which has been described as “normalized” after the acute phase of disease (Besche-Richard et al., 2014). A modulation by the SOA seems to be important here. Thus, a reduced N400 effect tended to be detectable with long SOAs, which, in contrast to short intervals, favor controlled search processes (i.e., using strategies such as prediction; Kuperberg et al., 2010), whereas short SOAs tended to lead to statistically normal or enhanced N400 effects (Wang et al., 2011; Mohammad and DeLisi, 2013). Also the presentation of homonyms in their sub-dominant meaning (e.g., *bank*: riverbank = sub-dominant; financial institution = dominant) led to a diminished N400 effect, which has been interpreted as a preferential preselection or a reduced suppression of dominant word meanings in persons with schizophrenia (Salisbury, 2010). This finding could reflect a reduced impact of semantic context, which can suppress dominant word meanings in specific circumstances (Heinz et al., 2019). Lastly, also the presentation of high-frequency words (i.e., words that occur frequently in a language) led to markedly large N400 amplitudes, indicating a disruption of their preferential activation (Condray et al., 2010).

##### 2.2.1.2 Lexico-semantics (production)

Verbal fluency (VF) tests require the production of as many correct words as possible in a short period of time. Thus, an underlying alternation between automatic spreading activation within the semantic network and active retrieval strategies using working memory and executive functions is assumed (Troyer et al., 1997; Turner, 1999; review see Henry and Crawford, 2005). Whereas semantic VF (i.e., the production of words belonging to a given semantic category) is thought to be based more on intact semantic memory functions and in particular on activation of language-relevant regions in the temporal area, phonemic VF (i.e., the production of words starting with a given letter) seems to depend more on frontal executive functions (Henry and Crawford, 2004; Baldo et al., 2006). In addition to the total number of words, the number and size of so-called clusters (i.e., the rapid production of usually related words) and switches (i.e., slow changes between mostly unrelated words) are commonly assessed (Gruenewald and Lockhead, 1980; Troyer et al., 1997; Ehlen et al., 2020).

**Figure 2:**
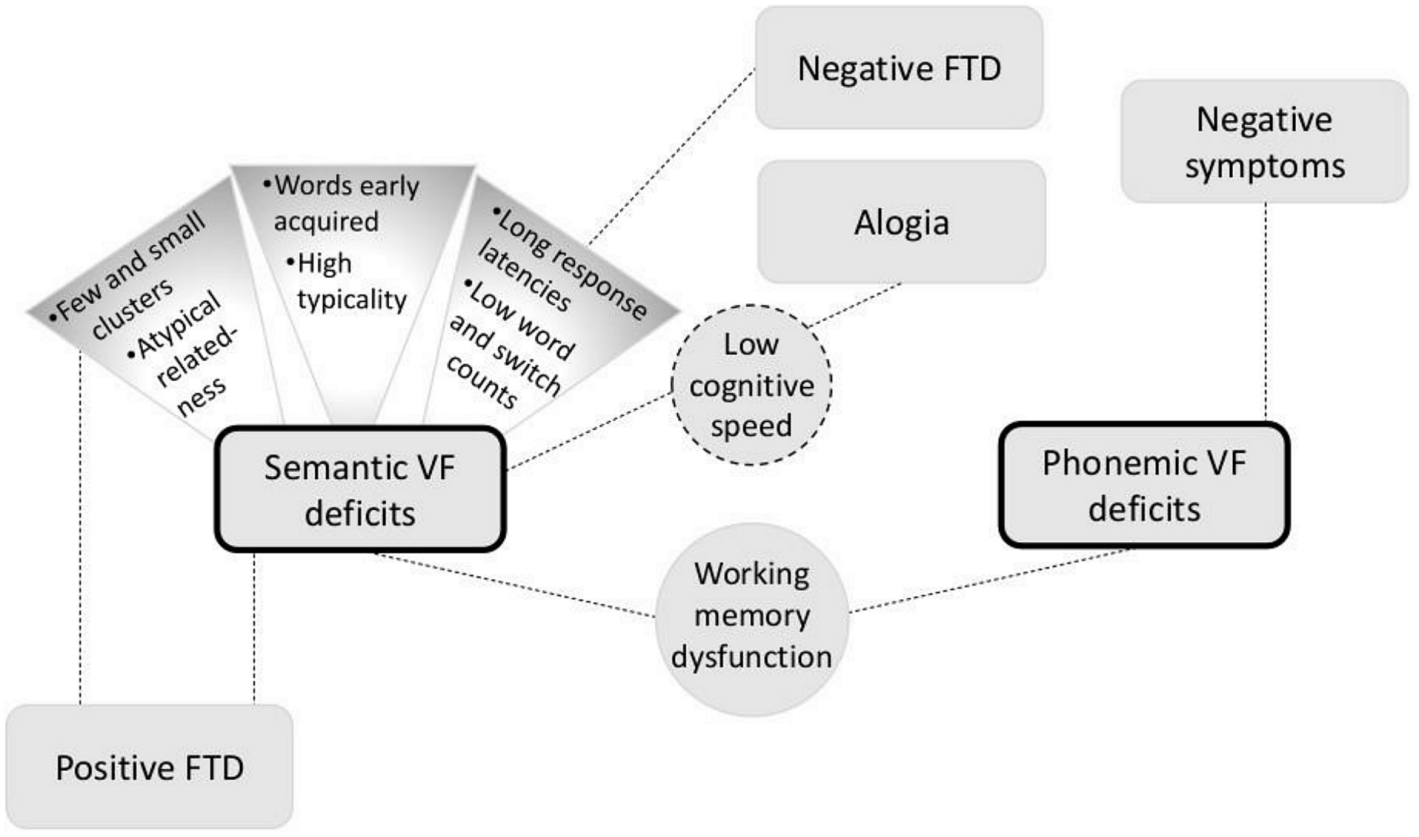
Illustration of associations between verbal fluency (*VF*) deficits and further findings including formal thought disorder (*FTD*) as suggested by current studies.

In persons with schizophrenia, deficits in semantic VF have been repeatedly shown with high effect sizes, whereas phonemic VF was little or not affected (Neill et al., 2014; Tan et al., 2015; meta-analysis see Knowles et al., 2010; Schaefer et al., 2013). As depicted in Figure 2, both semantic and phonemic VF deficits have been associated with working memory dysfunction (Ojeda et al., 2010). Phonemic VF deficits have additionally been related to negative symptoms (Ojeda et al., 2010). Deficits in semantic VF, on the other hand, have been associated with both positive FTD (Egeland et al., 2018) and—mediated by cognitive speed (as measured by the *Digit Symbol Substitution Test*; Wechsler, 1997)—alogia (Brébion et al., 2018) as well as with disease duration (Ojeda et al., 2010). Semantic tasks furthermore revealed above-average production of early acquired words with high typicality (expressed as percentage values based on normed data from categorization tasks) (Juhasz et al., 2012). Apart from that, in semantic tasks, persons with schizophrenia produced comparatively few and small clusters of semantically related concepts (Berberian et al., 2016; Tan et al., 2020), which differed in their semantic cluster patterns from those typically observed in healthy controls (Sung et al., 2012). Here, a lower number of related words was associated with positive FTD, whereas negative FTD were associated with increased response latencies (Docherty et al., 2011) as well as low word and switch counts (Egeland et al., 2018).

These results have been interpreted to support the presumed relationship between disorganized language and semantic memory structure disorders on the one hand, and negative FTD and executive dysfunction on the other (Docherty et al., 2011). Hypothetically, a negative correlation between switches in phonematic VF tasks and cerebellar GABA levels (quantified by magnetic resonance spectroscopy) in persons with schizophrenia may reflect a possible overinhibition within the prefronto-cerebellar system, which might normally contribute to the suppression of competing alternatives in VF tasks (Piras et al., 2019). In addition to the classic semantic and phonematic tasks, VF deficits also affected verb production (Badcock et al., 2011; Smirnova et al., 2017). Over a one-year course of illness, VF deficits appeared unchanged (Grimes et al., 2021) and seemed to persist across studies independently of other disease-related factors in persons with first psychotic episode, chronic schizophrenia, as well as their first-degree relatives (Tan et al., 2020).

Furthermore, pronounced deficits in picture naming tests for verbs and nouns as a function of picture complexity have been described (Kambanaros et al., 2010). Finally, categorical word production tasks yielded inconsistent results with evidence for the production of less typical responses by persons with schizophrenia (Brébion et al., 2010, but *cf.* Brébion et al., 2013) that could be related to thought disorganization (Brébion et al., 2013, but *cf.* Brébion et al., 2010). Affective flattening, on the other hand, has been associated with unimpaired use of typical words and interpreted as a possible inhibition of commission errors (Brébion et al., 2010, 2013). In this context, also neuroleptic effects have to be taken into account that cause secondary negative symptoms, particularly affective flattening and apathy, due to impairment of dopamine neurotransmission (Heinz et al., 1998).

#### 2.2.2 Syntax

Disturbances in the processing of complex syntax have been replicated several times in recent studies in the sense of decreasing sentence comprehension with increasing syntactic complexity (Dwyer et al., 2014; Tan et al., 2016; Çokal et al., 2019; Tan and Rossell, 2019; di San et al., 2022). Corresponding deficits have been associated with impairments in working memory (di San et al., 2022). However, given the suggested relationship between syntactic comprehension deficits and positive FTD (particularly derailment, circumstantiality, and loss of goal; Tan et al., 2016), syntactic processing deficits have been proposed as a subcomponent of positive FTD (Tan and Rossell, 2019; meta-analysis see Bora et al., 2019). It has been reported that such impairments are particularly evident in individuals with schizophrenia with FTD (Çokal et al., 2019), although they also occurred to a lesser extent in persons with schizophrenia without FTD, which may support a presumed continuum of context-dependent processing deficits in schizophrenia (Dwyer et al., 2014).

The current literature search identified no studies evaluating syntax production in individuals with schizophrenia, except for those comparing schizophrenia with bipolar disorder. The corresponding results are summarized in 2.5.1.

#### 2.2.3 Pragmatics

Pragmatics is traditionally distinguished from semantics and syntax by describing the use and comprehension of linguistic expressions in an interactional context that enables communication using common reference points including, e.g., abstract terms, idioms, and allusions (Morris, 1938). Up to 96% of individuals with schizophrenia have been found to exhibit deficits in subdomains of pragmatics (Bambini et al., 2016), while generally all domains of linguistic, extra-and paralinguistic, contextual, and communicative aspects of both comprehension and production may be affected (Colle et al., 2013). Corresponding deficits were associated with (socio)cognitive dysfunctioning (Bambini et al., 2016) and overall symptom severity (Colle et al., 2013). Logical reasoning, on the other hand, has also been suggested to be impaired, but studies are inconsistent (Gottesman and Chapman, 1960; Mujica-Parodi et al., 2000).

##### 2.2.3.1 Pragmatics (processing)

The process of contextual comprehension is conceivable as the ongoing computation of semantic relations between individual words and their matching against semantic memory with parallel combinatorial processing that ultimately enables the integration of lexico-semantic with syntactic and contextual information (Kuperberg et al., 2010). In persons with schizophrenia, depending on the complexity of the linguistic requirements, there could be an imbalance with a predominance of lexico-semantic matching to the detriment of combinatorial-integrative analysis, which could explain difficulties, e.g., with semantic ambiguities (Kuperberg et al., 2010).

Individuals with schizophrenia have classically been described to have an increased literal proverb understanding, traditionally labeled “concretistic” (Chapman, 1960), which has been replicated using metaphors (Deamer et al., 2019; review see Rossetti et al., 2018), idioms (review see Sela et al., 2015), or irony (review see Rapp et al., 2013). Here, the relevant context that indicates whether an utterance is to be taken literally or reflects a proverb may fail to impact on language processing (Heinz et al., 1996), in accordance with theories that precision of prior knowledge is reduced in psychotic states (Adams et al., 2016; Heinz et al., 2019).

Recent studies indicated low impairment if a choice answer was provided (Ketteler et al., 2012), whereas the formulation of an individual explanation—particularly of proverbs—led to significant impairments (Mercado et al., 2011; Bambini et al., 2020). Metaphor and humor comprehension seemed selectively preserved in individuals with first-episode schizophrenia (Pawełczyk et al., 2018), whereas individuals with a longer history of illness showed impairments in all domains of pragmatics (Pawełczyk et al., 2018). In this context, an association with a disease-related cognitive impairments was shown in persons with first psychotic episode (Perlini et al., 2018). With reference to social functions, proverb comprehension correlated with social cognition (Comparelli et al., 2020), and an association was shown between misinterpretation of metaphors and faulty evaluation of socially appropriate behaviors (Fukuhara et al., 2017).

Regarding the directionality of the disorders, individuals with schizophrenia were suggested to have intact bottom-up processing at the levels of (sub)-lexical sentence and discourse comprehension but impaired top-down processing (Stephane et al., 2014). Conversely, disturbances in the recognition of word relations (antonyms, homonyms, hyperonyms, and synonyms) were already detectable at the lexical level even without sentence context, and correlated with symptom severity (Ketteler et al., 2012).

Also at the sentence and discourse level, the N400 component usually shows larger amplitudes in the presence of contextual incongruence (Kutas and Federmeier, 2011). Individuals with schizophrenia exhibited an early component during which the N400 depended exclusively on contextual congruence and a later component that depended exclusively on semantic associations, whereas healthy individuals showed parallel processing at both levels (Ditman et al., 2011). This was attributed to an impairment of initial activation and later inhibition of semantic associations, whereby individuals with schizophrenia might use the context initially but fail to maintain it when conflicting semantic associations arise (Ditman et al., 2011). Moreover, a reduced N400 effect when lexical associations were not available despite congruent contextual information pointed to impaired discourse comprehension due to excessive reliance on semantic associations (Swaab et al., 2013).

##### 2.2.3.2 Pragmatics (production)

The formation of linguistic references, e.g., between mentioned characters, temporal, causal, and local information plays a crucial role for interference-free communication and corresponding impairments are called disorders of referential coherence (Ditman et al., 2011). Coherence implies a common frame of reference between the interlocutors. In persons with schizophrenia, disorders of referential coherence can manifest in disjointed speech flow with diminished thematic references between words and sentences, and also in the violation of conversational rules like quantity, relevance, regard of shared vs. new information etc. (Grice, 1975; Corcoran and Frith, 1996; Brüne and Bodenstein, 2005; Kuperberg, 2010a,b). Coherence in relation to the overarching content and joint reference frame between sentences or parts of speech, can be distinguished in part from cohesion, which rather refers to the surface structure of a sentence. Cohesion results from the immediate establishment of connections between concepts through the use of appropriate conjunctions, pronouns, substitutions, etc. (Halliday and Hasan, 1976).

In recent studies, individuals with schizophrenia and clinical FTD showed failures in the use of referential pronouns (Sevilla et al., 2018), unclear linguistic references (Çokal et al., 2018), as well as contextually inappropriate word substitutions and misinterpretations in repetition tasks, most likely due to impaired word access or impaired contextual integration (Dwyer et al., 2014). At the communication level, there were difficulties in relating to the current communication topic and pronounced disturbances in language production in informal vs. formal contexts (Colle et al., 2013). Also, there were associations between reduced use of conjunctions and reduced capacity for metacognition (Buck et al., 2015), and between impaired Theory-of-Mind and reduced alignment of word use with the communication partner (Dwyer et al., 2020) (here, alignment means that the speaker and the listener align their linguistic representations, thus facilitating communication). In contrast to this observation, unimpaired alignment was described in another study (Sharpe et al., 2022), and social interactions appeared to enhance reference formation, semantic cohesion, and contextualization (Saavedra, 2010).

With regard to the ability to infer the either concrete or abstract meaning of an utterance according to context, production might be less impaired than comprehension: For example, the use of abstract expressions in free speech was only slightly reduced in individuals with schizophrenia despite impaired metaphor comprehension (Elvevåg et al., 2011). Also the completion of given phrases was unimpaired despite a slowing associated with negative symptomatology (Pesciarelli et al., 2014). In contrast, marked deficits in the domain of irony have been described in patients with chronic schizophrenia not only in comprehension but also in production (Colle et al., 2013).

*In summary*, linguistic analyses delineated impairments on the levels of lexico-semantics, syntax and pragmatics. Results on lexico-semantic processing showed hyperpriming likely associated with positive FTD, hypopriming, and mainly reduced N400 effects. Regarding word production, especially deficits in semantic VF point to a disease-related dysfunction of semantic memory, which seemed persistent, could intensify as a function of disease duration and was also observed in first-degree relatives. Corresponding deficits appear to affect mainly the automatic activation of closely related items and may have a greater impact on neural connections formed in the later phase of word acquisition.

Individuals with schizophrenia typically exhibit deficits in processing complex syntax. This has been associated with working memory deficits and positive—but not negative—FTD and may also occur in the absence of FTD.

Concerning pragmatic language comprehension, the long-standing finding was confirmed that explaining inferential meaning (e.g., in idioms or irony) is impaired. This could particularly affect individuals with disease-related cognitive impairments. Corresponding deficits could be closely related to impairments in social cognition, suggesting a common underlying alteration in context-dependent understanding of semantic expressions. This could be attributed to predominant processing based on semantic relations with reduced contextual integration, possibly due to impaired top-down processing. At the level of pragmatic language production, especially impaired use of coherence and referential cohesion was found, which may interfere with social communication.

### 2.3 Findings from natural language processing

With the aim of improving the objectivity of language-related findings, various NLP approaches have been developed in recent years. In particular, these have been investigated for their utility in characterizing psychosis-typical language features and for their predictive value for the development of psychotic episodes (reviews see Corcoran et al., 2020; Hitczenko et al., 2021). Machine learning has been widely used for this purpose (Corcoran et al., 2018; Perlini et al., 2018; Rezaii et al., 2019; Girard et al., 2021; Sarzynska-Wawer et al., 2021; Voppel et al., 2021; Ziv et al., 2022). In the following, NLP findings related to individuals diagnosed with psychotic disorders are presented first. Findings on early diagnosis and comparative studies are summarized in the subsequent sections.

#### 2.3.1 NLP-findings in persons diagnosed with psychotic disorders

After Latent Semantic Analysis (*LSA*) (see Box 2) was first used to distinguish individuals with schizophrenia/schizoaffective disorder from healthy persons on the basis of reduced discourse coherence with 80% accuracy (Elvevåg et al., 2007), the method has been widely used and adapted (reviews see Corcoran et al., 2020; Hitczenko et al., 2021). For example, using *CoVec* (see Box 2) resp. *LSA*, associations have been demonstrated between reduced semantic coherence in VF tests and derailment, tangentiality (Pauselli et al., 2018), severity of psychotic symptoms, and impairments in psychosocial functioning (Holshausen et al., 2014). Also in terms of reduced coherence, increased variance of word relatedness in free speech has been demonstrated in individuals with schizophrenia spectrum disorder using *Word2Vec* (see Box 2), allowing discrimination from healthy individuals with approximately 85% accuracy even in the presence of low positive symptomatology (Voppel et al., 2021). In an own investigation, 60% of patients with non-affective psychoses and clinical positive FTD classified correctly by automatically derived coherence metrics; however, the addition of ambiguous referential markers and neologisms improved the model (Just et al., 2023). The newer analysis methods *BERT* (Devlin et al., 2019) and *ELMo* (Peters et al., 2018) incorporate more contextual information into their analyses (see Box 2). Using the *BERT*, decreased question-answer coherence was found, indicating increased tangency, and based on frequent use of incomplete words, assignment to a group with schizophrenia/schizoaffective disorder was made with 90% accuracy, although FTD was clinically detectable in only 20% (Tang et al., 2021). Using the *ELMo*, texts could be assigned to persons with schizophrenia with over 80% accuracy, especially when many words with low semantic content (e.g., “somehow,” “well”), few positive emotions, or many spiritual words were used (Sarzynska-Wawer et al., 2021). Worth mentioning, in the same study, correct assignment was achieved with over 70% accuracy using the *TLC*. Moreover, persons with (non-acute) schizophrenia were identified by the production of more words and more errors, with both variables correlating with positive FTD and with positive symptoms in the *PANSS* without correlation with negative symptoms (Tan et al., 2021). Similarly, exploratory cohesion analyses with *Coh-Metrix* (McNamara et al., 2014, see Box 2) indicated an association between increased use of pronouns, causal and temporal conjunctions in persons with schizophrenia and linguistic disorganization (Mackinley et al., 2021). On the other hand, part-of-speech tagging was able to assign texts to persons with schizophrenia with 80–90% prediction accuracy due to, e.g., reduced verbs, adverbs, and adjectives, more diverse lexemes, and more frequent use of the first person singular (Ziv et al., 2022). This has been interpreted as less complex, more associative, and more self-centered language and may be indicative of poverty of content and reduced cohesion. Regarding decreased linguistic cohesion (determined by *Coh-Metrix*) as a marker for negative FTD, a statistical path model implied that impaired causal/intentional cohesion—partly direct, partly mediated by decreased self-reflectivity (as measured by the *Metacognition Assessment Scale-Abbreviated*; Lysaker et al., 2005)—may lead to simplified language that is difficult for the other person to understand (García-Mieres et al., 2020). In addition, both using *Coh-Metrix* (Mackinley et al., 2021) and the *Analytic Thinking Index* (Silva et al., 2021), persons with first-episode schizophrenia were shown to have greater narrativity (i.e., episodic, narrative, and less formal language style), which correlated with disorganized thinking in the *PANSS* and the *TLI* and may indicate a lower capacity for hierarchical structuring of thoughts. Finally, emotion-related autobiographical texts have been attributed to individuals with schizophrenia on the basis of lower expressivity and complexity, more self-reference, and more repetition especially when referring to anger and joy (Hong et al., 2015).

**BOX 2 Overview of NLP coherence and cohesion analysis methods.**

Coherence analyses (LSA, CoVec, Word2Vec)

Software programs such as *Latent Semantic Analysis* (*LSA*) (Landauer and Dumais, 1997, *cf.* Elvevåg et al., 2007), *Word2Vec* (Mikolov et al., 2013) and *CoVec* (Covington, 2016) use corpus-based contextual word frequency distributions represented as a high-dimensional vector space such that word similarities are computed based on the respective angles between the vectors. This enables the quantification of the similarity of all individual produced words as well as whole sentences to each other (discourse coherence).

*BERT*

The *Bidirectional Encoder Representations from Transformers* (*BERT*) (Devlin et al., 2019) is pre-trained using a comprehensive corpus and incorporates the respective linguistic context into the lexical analysis. Two methods are used here: (1) masked language modeling, where 15% of words are randomly removed from an input text and BERT predicts these words, (2) next sentence prediction, where given two sentences, BERT predicts the probability that the second sentence directly follows the first (Tang et al., 2021).

*ELMo*

*Embeddings from Language Models* (*ELMo*) (Peters et al., 2018) uses so-called embeddings. Here, each word/statement is assigned to a vector whose values are derived from semantic and syntactic properties, so that words/statements with similar meaning are similarly represented thereby enabling content-based text comparisons. Here, the system is trained similarly to a neural network (Sarzynska-Wawer et al., 2021).

*Coh-Metrix*

The *Coh-Metrix*-System (McNamara et al., 2014) is a web-based automatic speech analysis software that includes 108 indices in 11 categories. Here, extensive, automated computation of word-and text-related variables occurs, ranging from basic processes (typically at the word or sentence level) to complex processes (including organization, narrativity, and cohesion within and between sentences and text passages).

*In summary*, NLP studies particularly showed reduced semantic and discourse coherence in persons with schizophrenia, which seems to be related to positive FTD, but is also detectable in clinically low-grade FTD. In this regard, high discrimination accuracy between individuals with and without psychotic disorders has been repeatedly shown. Also the production of incomplete and content-poor words, low use of verbs, adverbs, and adjectives, reduced expressiveness and complexity, a less formal but more narrative style of language, and increased self-reference seem to be typical of schizophrenia. Finally, the production of more words overall (especially conjunctions and pronouns) and more errors could be specifically related to positive symptoms.

### 2.4 Early detection and predictive value

The predictive value of linguistic analyses was first demonstrated in adolescents at clinical high risk with a specific association between “illogical” thinking (assessed as “inappropriate” causal or noncausal utterances or statements which are simultaneously made and refuted by the *Kiddie Formal Thought Disorder Rating Scale, K-FTDS*; Caplan et al., 1989), but see for inconsistent results (Gottesman and Chapman, 1960; Mujica-Parodi et al., 2000), poverty of content (as assessed by the *K-FTDS*), as well as decreased referential cohesion (as assessed by a modified analysis of cohesion; Halliday and Hasan, 1976) and the manifestation of psychosis (Bearden et al., 2011). Comparably, an association between disorganization and manifestation of psychosis has been repeatedly shown in adults at clinical high risk using clinical scales (e.g., Demjaha et al., 2012; DeVylder et al., 2014; review see Oeztuerk et al., 2022). Studies at the levels of lexico-semantics, syntax, and pragmatics revealed findings in individuals at high risk that were comparable to those of persons with schizophrenia, and in some cases, associated with an increased risk for manifestation of psychosis. Comparable to persons with schizophrenia (Wang et al., 2011; Mohammad and DeLisi, 2013), in persons at clinical high risk, the N400 effect in priming studies was largely intact at short SOAs but markedly diminished at long intervals (Lepock et al., 2019), which was associated with a decrease in global functioning levels (Lepock et al., 2022). This was interpreted in terms of intact early activation of related concepts but poor maintenance of such activation and suggested as a possible neurophysiological biomarker for schizophrenia risk (Lepock et al., 2019). Individuals at clinical high-risk furthermore showed marked impairment in semantic but not phonematic VF (Becker et al., 2010; Magaud et al., 2010), predictive of transition to manifest psychosis (Becker et al., 2010). On the other hand, an association between decreased phonematic VF and transition to schizophrenia has also been demonstrated (Pawełczyk et al., 2021). Underlying the VF deficits may be a specific impairment in word selection despite intact single-word retrieval, which in individuals at ultra-high-risk correlated with both disorganized and negative symptoms (Vargas et al., 2018).

More equivocal findings emerged from comprehensive studies of pragmatics: Here, individuals at ultra-high-risk showed significant impairments in understanding inferential meanings, in discourse analysis, and in the socio-emotional domain, but better performance in metaphor explanation and in the cognitive domain than individuals with first psychotic episode (Pawełczyk et al., 2019). However, transition to schizophrenia was specifically associated with impairments in humor comprehension and metaphor explanation (Pawełczyk et al., 2021).

Also NLP-studies indicated some similarities to the above findings in individuals with schizophrenia: Thus, correlations were reported between low semantic coherence and positive FTD (tangentiality, derailment, and circumstantiality) as well as between reduced lexico-semantic complexity and negative FTD in individuals with clinical high risk using the *BERT* (Bilgrami et al., 2022).

Using *word2vec*, individuals at clinical high risk showed reductions in coherence and on-topic scores (as a measure of tangentiality) comparable to those of individuals with first psychotic episode (Morgan et al., 2021). That said, this pattern was most apparent in picture descriptions (rather than during free speech) and was no longer significant when controlling for IQ and schooling (Morgan et al., 2021). Beyond that, reduced cohesion in the form of reduced word stem overlap was detectable by *Coh-Metrix* in individuals at ultra-high-risk, which was associated with an increase in positive, negative, and disorganized symptoms in the *SIPS* as well as impairments in verbal learning (Gupta et al., 2018). Furthermore, individuals at genetic high risk for schizophrenia showed increased atypical lexical associations, which correlated with higher schizotypy scores, but occurred in only one of five picture description tasks (Manschreck et al., 2012). In addition, building on a proof-of-principle study (Bedi et al., 2015) transition from a prodromal phase to a psychotic episode was predicted with 80% accuracy by *LSA* and part-of-speech tagging, with primarily lower semantic coherence and lower use of possessive pronouns identified as critical distinguishing features (Corcoran et al., 2018). Here, the computational linguistic findings correlated with illogical thinking, content poverty (both clinically assessed with the *K-FTDS*), and low referential cohesion (Corcoran et al., 2018). In contrast, a joint analysis of *LSA*-based semantic coherence, part-of-speech tagging, brain structural findings, and functional connectivity did not allow discrimination between individuals at clinical high-risk and healthy individuals (Haas et al., 2020). Similarly, during treatment of an acute psychotic episode (in schizophrenia, bipolar disorder, major depression, or a related disorder), no predictive value of NLP-based lexical, sentence coherence, or disfluency measures could be demonstrated in relation to positive or negative symptoms (Girard et al., 2021). Yet, correlation were shown between positive symptoms and increased use of perceptual process words indicative of perceptual distortion, as well as between negative symptoms and decreased use of relative words suggesting poverty of speech, increased negative emotion words, and error corrections (Girard et al., 2021).

As a further NLP approach, speech graphs were investigated with regard to their predictive value for psychotic disorders. In this method, which is based on graph theory, a network-like modeling of individual linguistic utterances is carried out independently of semantic or syntactic features (Mota et al., 2012; see Box 3). The performance of the method has been evaluated as exceptionally high, but with uncertainty as to which anomalies it reflects and how valid it is (Hitczenko et al., 2021). Compared with individuals at clinical high risk, the connectivity of speech graphs from picture descriptions was reduced (indicated by lower LCC, LCCr, LSCr; see Box 3) in individuals with a first psychotic episode and correlated with negative FTD in the *TLI* (Morgan et al., 2021; Spencer et al., 2021). Notably, LSC and LSCr were also significantly reduced in those who developed psychosis within the following 7 years, suggestive of a relationship between speech connectedness and transition to psychosis (Morgan et al., 2021; Spencer et al., 2021). Finally, the NLP methods *Vector Unpacking* and *Latent Context Analysis* (see Box 3) were able to achieve a predictive ability of 90% for the later manifestation of schizophrenia in persons in the prodromal phase. These findings were based on low semantic density (as a measure of content poverty), which correlated with negative symptoms, and increased references to voices, whispers, and sounds that correlated with positive symptoms (Rezaii et al., 2019). Since the vector-based computation was strongly correlated with the assessment of semantic density by human raters, this method was also found to be particularly promising (Hitczenko et al., 2021).

**BOX 3 Method descriptions of speech graphs, vector unpacking, latent context analysis.**

Speech Graphs

The individual words spoken in a text are each mapped only once in their lexeme form (as so-called nodes) and connected to each other in their temporal sequence by directed graphs (as so-called edges) (Mota et al., 2012) using *SpeechGraphs software* (Mota et al., 2014). This results in both single and multiple connections, including circuit connections, whose length and the number of nodes and edges are interpreted as measures of speech connectivity. The most important metrics are defined as:

- LCC (= largest connected component) is the sum of the nodes within the largest open connected component of the graph in which the nodes are connected by at least one directed path (Mota et al., 2012)
- LSC (= largest strongly connected component) is the set of nodes within the largest closed component of the graph in which each pair of nodes is connected by a direct or indirect path and thus mutually reachable, i.e., node “a” reaches node “b” and node “b” reaches node “a” (Mota et al., 2012)

In order to additionally calculate how close the degree of connectedness of the graphs is to random, the values LCC and LSC are additionally determined for random mixtures of the words, and the values LCCr and LSCr are defined as the ratio between original connectedness and random connectedness. Values closer to 1 mean that the spoken language structure is closer to a random structure. The values are interpreted as:

- LCCr: A value close to 1 indicates random connections with a low degree of goal directedness (Palaniyappan et al., 2019)
- LSCr: A value close to 1 indicates a lack of referential ties, i.e., a collection of randomly connected words (Palaniyappan et al., 2019).

Vector unpacking

The content words of a sentence are represented as multidimensional vectors (here by *Word2Vec*) based on their semantic meaning and calculates the relationship between the meaning-bearing vectors and all words in a sentence (Rezaii et al., 2019).

Latent context analysis

Individual language vectors are compared with those of an extensive corpus, so that not only explicit but also implicit content can be assessed (Rezaii et al., 2019).

*In summary*, primarily disorganization, some degree of illogical thinking, poverty of content, and reduced referential cohesion as assessed by clinical scales as well as impaired semantic and phonemic VF, and difficulties in understanding humor and explaining metaphors may be associated with conversion from the at-risk state to first psychotic episode. NLP studies furthermore pointed to reduced semantic coherence (possibly best detectable with the *BERT*), reduced connectivity of speech graphs, reduced semantic density, and increased references to auditory sensations as possible predictors of conversion to psychosis.

### 2.5 Comparative studies

#### 2.5.1 Schizophrenia compared to bipolar disorder

Already Bleuler pointed out the necessity of distinguishing between disjointed speech in persons with schizophrenia and severe forms of flight of ideas with accelerated thinking in persons in manic episodes (Bleuler, 1911b). Andreasen and Grove showed a preponderance of (prognostically unfavorable) negative FTD in individuals with schizophrenia compared with tendentially stronger and transient positive FTD in individuals with mania (Andreasen and Grove, 1986).

Recent comparative studies that used clinical scales have supported the hypothesis of more frequent negative FTD in persons with schizophrenia and also showed persistent positive FTD to occur more frequently in them than in persons with bipolar disorder (e.g., Wilcox et al., 2012; meta-analysis see Yalincetin et al., 2017). However, dimension reduction procedures argued for the conceptualization of FTD as a continuum with a highly specific distribution of the factors “Verbosity,” “Poverty of Speech,” and “Disorganization” with specifically increased verbosity and overall more frequent FTD in first-time manic episodes vs. first manifestation of schizophrenia (45% vs. 19%) (Roche et al., 2015, *cf.* Cuesta and Peralta, 2011). When groups were matched for overall levels of positive FTD and cognitive performance, differences consisted solely of more poverty of content of speech and perseveration in the group with schizophrenia (Kircher et al., 2022).

At the lexico-semantic level, individuals with schizophrenia made more errors than those with bipolar disorder when assessing word associations (e.g., “honey” and “stings” both counting as associated with “bee”), with erroneous associations between unrelated terms being related to FTD severity and missing associations being related to negative symptoms (Jamadar et al., 2013). When explicitly asked to judge word relatedness, preserved N400 effects were measured in individuals with schizophrenia (Ryu et al., 2012; Wang et al., 2020), with decreased N400 amplitudes overall (Wang et al., 2020) or specifically for unrelated words (Ryu et al., 2012). In contrast, conflicting results indicated comparatively large N400 amplitudes for related words with reduced N400 effect (Ryu et al., 2012) or overall slightly reduced N400 amplitudes with preserved N400 effect (Wang et al., 2020) in persons with bipolar disorder. Moreover, a preceding presentation of homophones elicited a magnification of the N400 congruency effect in people with schizophrenia, whereas it was diminished in healthy individuals and those with bipolar disorder (Raucher-Chéné et al., 2019).

Compared to individuals with bipolar disorder, word production in semantic (but not phonemic, Meesters et al., 2013) VF tasks was reduced in individuals with schizophrenia (Esan et al., 2020)—especially in the acute phase, but also in remission (Ceylan et al., 2020) and in elderly (Meesters et al., 2013). The former group did not differ from healthy controls (Ceylan et al., 2020) or performed worse (Meesters et al., 2013; Esan et al., 2020).

At the syntactic level, both individuals with schizophrenia and bipolar disorder made more comprehension errors than healthy controls (Perlini et al., 2012). Here, individuals with schizophrenia produced shorter sentences, made more paragrammatic errors, and exhibited lower syntactic completeness (Perlini et al., 2012). The amplitude of the ERP component P600, which is associated with a second-parse attempt to understand syntactically invalid sentences (*cf.* Kuperberg et al., 2010), was reduced both in individuals with schizophrenia and bipolar disorder, which was associated with a reduction in the P600 effect, pointing to a failure of contextual integration in both disorders (Lee et al., 2016).

At the discourse level, coherence was decreased in persons with schizophrenia compared to healthy individuals, especially in the case of severe overall symptoms, whereas no differences were found compared to individuals with bipolar disorder (who themselves did not differ significantly from healthy individuals) (Perlini et al., 2012).

Speech graphs in particular have been used as NLP methods to differentiate between individuals with schizophrenia and bipolar disorder. In a first pilot study, when applied to dream reports, they showed a discriminability between both groups with more than 90% accuracy (Mota et al., 2012). In particular, decreased connectivity was described in persons with schizophrenia (Mota et al., 2012; Palaniyappan et al., 2019), as well as increased knots and edges and frequent changes in contextual references in persons with mania (Mota et al., 2012). Exclusively in persons with schizophrenia, associations were shown between low LSCr (i.e., lower referential ties, see Box 3) and global functional level, and between low LCC and LCCr (i.e., lower speech connectedness, see Box 3) and decentralization of the core brain hubs in fMRI (Palaniyappan et al., 2019). This was interpreted as a biological marker of cerebral dysconnectivity that could condition speech dysconnectivity (Palaniyappan et al., 2019). However, the authors found no correlations with clinical measures of FTD. It was furthermore possible to distinguish between individuals who developed schizophrenia or bipolar disorder 6 months after initial manifestation of a psychotic syndrome with very high accuracy on the basis of a “disorganization index” derived from LCC and LSC, which correlated with negative symptoms in the *PANSS* and was more pronounced in the antecedent of schizophrenia (Mota et al., 2017). Importantly, the results were obtained exclusively from dream reports and negative picture stories, but not from the history interview or from memory descriptions. In contrast to the above results (Meesters et al., 2013; Esan et al., 2020), application of speech graph analysis to VF tasks showed no significant differences between the two clinical groups, although individuals with schizophrenia presented the expected differences from healthy individuals in terms of lower nodes and edges (Chrobak et al., 2022).

#### 2.5.2 Schizophrenia compared to schizoaffective disorder

No relevant differences were found between individuals with schizophrenia and schizoaffective disorder using the *TLC* (Wilcox et al., 2012) or comprehensive linguistic assessments (Pantano et al., 2016) and in many of the studies mentioned above, mixed cohorts were summarized (i.e., Bowie et al., 2011; Docherty et al., 2011; Mercado et al., 2011; Becker et al., 2012; Juhasz et al., 2012; Wilcox et al., 2012; Stephane et al., 2014; Tan et al., 2014, 2015, 2016; Smirnova et al., 2017; Muralidharan et al., 2018; Tan and Rossell, 2019; García-Mieres et al., 2020; Girard et al., 2021; Grimes et al., 2021; Tang et al., 2021; Voppel et al., 2021; Chrobak et al., 2022; Sharpe et al., 2022). Nonetheless, a study of communication failure found stronger deficits in persons with (non-acutely psychotic) schizophrenia than with schizoaffective disorder (Docherty et al., 2013). Here, more than 50% of the communication dysfunctions, especially in the group with schizophrenia, were explained by combined as well as single deficits in the areas of neurocognition, emotional perception, and Theory of Mind (Docherty et al., 2013).

#### 2.5.3 Schizophrenia compared to depressive disorder

Individuals with schizophrenia did not differ from (non-age and education matched) individuals with depressive disorders in basic semantic-lexical language skills and showed better comprehension of emotional prosody (Pawełczyk et al., 2020). Yet, in pragmatics, they showed significantly poorer abilities in inferring implicit meanings, understanding humor and metaphors, and discourse comprehension, regardless of symptom severity (Pawełczyk et al., 2020). Beyond that, exclusively in individuals with schizophrenia, a pronounced affective reactivity of language was demonstrable in the form of reduced information reference in negative versus positive valence of topic (Rubino et al., 2011) and in the form of a general increase in FTD when speaking about social relations (Cohen et al., 2014). Fittingly, in persons with schizophrenia, negative but not positive or neutral sentence endings resulted in larger N400 amplitudes than in persons with depression/dysthymia, who in turn did not differ from healthy individuals (Klumpp et al., 2010).

#### 2.5.4 Comparison schizophrenia, bipolar disorder, schizoaffective disorder, affective disorder

Comparative studies that collectively examined FTD in cohorts with schizophrenia, manic and depressive episodes in affective and schizoaffective disorders using the *TLC* (Cuesta and Peralta, 2011), *TALD* (Kircher et al., 2014), or *SAPS*/*SANS* (Stein et al., 2022) confirmed a transdiagnostic occurrence of FTD with highly specific distribution of distinguishable symptom domains. Specifically, symptoms attributed to the factors „Objective positive “(e.g., derailment, crosstalk, dissociation of thinking, tangentiality, and logorrhea) (Kircher et al., 2014) or “Disorganization” (e.g., derailment, loss of goal, tangentiality, circumstantiality, and illogicality) and “Verbosity” (e.g., clanging, pressure of speech, echolalia, incoherence, and neologisms) (Cuesta and Peralta, 2011) were most pronounced in individuals with manic episodes. In contrast, symptoms attributed to the factors “Negative FTD” (e.g., poverty of thought, inhibited thinking, dysfunction of thought initiative and intentionality, poverty of speech, and slowed thinking) (Kircher et al., 2014) or “Poverty of Speech” (e.g., poverty of content of speech, poverty of speech, and perseveration) (Cuesta and Peralta, 2011) were particularly pronounced in persons with schizophrenia (Cuesta and Peralta, 2011). In addition, frequent “Idiosyncratic Speech” (e.g., word approximations, stilted speech, and neologisms) and “Disorganization” have been described here (Cuesta and Peralta, 2011). Persons with depressive episodes showed both highest scores in “Subjective negative FTD” (e.g., poverty of thought, inhibited thinking, dysfunction of thought initiative and intentionality) (Kircher et al., 2014) and lowest in “Disorganization” and “Verbosity” (Cuesta and Peralta, 2011).

Three factors were identified transdiagnostically with a differential correlation between (1) “Disorganization” and volume reduction in the left temporooccipital language region (comprising parts of the gyrus angularis and the middle occipital gyri) as well as the fractional anisotropy (FA) in the right cingulate and inferior longitudinal fasciculus, between (2) “Emptiness” and volume reduction in the hippocampus and thalamus, and between (3) “Incoherence” and FA in the right cingulate/hippocampal area and reduced FA of the anterior thalamic radiation (Stein et al., 2022).

*In summary*, the current body of research provides evidence for a transdiagnostic occurrence of clinically detectable FTD in persons with schizophrenia, bipolar, schizoaffective, or affective disorder. Individuals with manic episodes seem most prone to FTD with disease-nonspecific positive FTD (especially verbosity). High discriminability between individuals with schizophrenia and manic episodes was reached on the basis of disease-specific poverty of speech in schizophrenia with reduced word production, shorter sentences, and lower syntactic completeness. Moreover, persons with schizophrenia exhibited idiosyncratic speech and (nonspecific) disorganization. In addition, high discriminatory and predictive power could be achievable on the basis of reduced connectivity of speech graphs in individuals with schizophrenia. Strong overlap of FTD symptoms was reported between persons with schizoaffective disorder and schizophrenia, although the latter may present with more communication disturbances. Compared to individuals with depressive disorder, persons with schizophrenia may especially show impairments in pragmatics and an intensification of FTD typical of schizophrenia during socio-emotional stress.

## 3 Discussion

The present review focuses on FTD and investigations at the linguistic levels of lexico-semantics, syntax, and pragmatics in persons with schizophrenia. The reviewed literature confirms that individuals with schizophrenia exhibit numerous linguistic alterations, detectable by clinical scales as well as by more specialized linguistic analyses. We will critically discuss these findings with respect to their theoretical and clinical implications.

At the lexico-semantic level, several priming studies in persons with schizophrenia reported hyperpriming of semantically related items (Safadi et al., 2013; Kuperberg et al., 2019; Almeida and Radanovic, 2021), which has been associated with positive FTD (e.g., Safadi et al., 2013; Kuperberg et al., 2019). An underlying excessive activation of semantic neighbors (Brown and Kuperberg, 2015) and—with respect to neurobiological correlates—a dysfunction of inhibitory interneurons (Almeida and Radanovic, 2021) have been suggested. However, in view of the also frequently reported hypopriming (Niznikiewicz et al., 2010; Besche-Richard et al., 2014; Sass et al., 2014; Tan et al., 2015), the overall results appear inconclusive: Hypopriming has been interpreted in terms of executive dysfunction or semantic memory disorganization leading to impaired contextual integration (Tan et al., 2015; *cf.* Kuperberg, 2010a,b; Almeida and Radanovic, 2021). In this context, results from N400-studies hint at a potentially important effect of the experimental setting, in that reduced N400 effects both in persons diagnosed with schizophrenia (Wang et al., 2011; Mohammad and DeLisi, 2013) and in those at high risk (Lepock et al., 2019, 2022) were observed particularly when the interstimulus interval (SOA) was long. Since these long SOAs favor the use of controlled semantic search strategies (Kuperberg et al., 2010), automatic activation could be overactive (*cf.* Spitzer, 1993), while controlled access to semantic memory may be disrupted, hypothetically reflected by low N400 effects. On the other hand, a reduction in N400 effect was also observed in one study at short SOA and was associated with more severe positive symptoms (Besche-Richard et al., 2014). Regarding the comparison of persons with schizophrenia versus bipolar disorder, findings of decreased N400 amplitudes (Ryu et al., 2012; Wang et al., 2020) in persons with schizophrenia may further support a specific reduction in the differentiation between semantically related and unrelated items in the sense of a lowered signal-to-noise ratio among persons with schizophrenia. Interestingly, one study observed comparably large N400 amplitudes upon the presentation of high frequency words (Condray et al., 2010), which usually evoke a low N400 amplitude in control subjects (the so-called frequency-effect). The latter is thought to reflect an enhanced processing of words in a given context based on their familiarity (Kutas and Federmeier, 2011). Taken together, the above results could indicate, that under all these circumstances, context-related information may fail to prime specific semantic memory, or that memory structure itself may be altered. The hypothesis of reduced effects of context in psychosis is supported by the observation of a preferential preselection of homonyms in their dominant meaning (Salisbury, 2010), which can be explained as an understanding of the dominant word meaning to the expensive of the context-dependent inferential meaning (Heinz et al., 1996). Therefore, future priming and N400 studies controlling for both SOAs and symptomatology should also assess semantic memory structure as well as specific effects of sematic and spatial context on behavior.

In terms of word production, repeated findings of semantic VF deficits that precede the transition to psychosis (Becker et al., 2010), persist or exacerbate during the course of the illness (Besche-Richard et al., 2014; Tan et al., 2020; Grimes et al., 2021), and occur in first-degree relatives (Tan et al., 2020), suggest a link between altered priming within the semantic memory network or an altered network connectivity and a predisposition to schizophrenia. Indeed, reduced semantic connectivity was indicated by reduced coherence during semantic VF (Pauselli et al., 2018) as well as diminished clusters (Berberian et al., 2016; Tan et al., 2020) with atypical word relatedness (Sung et al., 2012). Importantly, the former was linked to clinically observable derailment and tangentiality and the latter to both positive (Docherty et al., 2011; Egeland et al., 2018) and negative (Brébion et al., 2018) FTD. In this context, the finding of an above-average production of early acquired words (Juhasz et al., 2012) could indicate a specific alteration of semantic network connections formed later during learning history. The preferential production of early acquired items in the general population (the co-called age-of-acquisition effect; for reviews see, e.g., Juhasz, 2005; Elsherif et al., 2023) could result from higher semantic network plasticity during early compared to later word acquisition, leading to more connections, more central positions, and easier access (Elsherif et al., 2023). In healthy individuals, word acquisition during individual development is accompanied by a continuous increase in gray matter density within temporo-parietal regions relevant for semantic and syntactic processes (Richardson et al., 2010). Thus, assuming a disease-associated impairment in the ability to use context (Heinz et al., 1996; Brown and Kuperberg, 2015), impaired context-based learning (Heinz et al., 2019) could contribute to reduced connectivity of late acquired words within the semantic network and their reduced retrieval. It thus seems conceivable that diminished semantic connectivity is an essential prerequisite for less conventionally structured thinking and may at least partially be present even before the first psychotic episode. On a similar note, semantic coherence was also reduced during free speech (Voppel et al., 2021), where it could be a predictor of transition from risk status to psychosis (Corcoran et al., 2018).

Yet, a creative aspect of language production should also be considered: Unusual word associations could be shaped by psychotic experiences that are difficult to verbalize. This could influence association-based responses especially in the case of negative affective content (e.g., related to trauma).

Finally, the effects of cognitive speed (Brébion et al., 2018) and working memory (Ojeda et al., 2010) on VF performance in individuals with schizophrenia need to be taken into account. These are primarily expected to cause prolonged (Docherty et al., 2011) and diminished switches (Egeland et al., 2018), which have been association with negative FTD. Reduced attention (Nagels et al., 2016; Ucok et al., 2021; Oeztuerk et al., 2022) could additionally contribute to reduced VF performance. Similarly, impaired working memory or cognitive load due to the complexity of the items to be retrieved could also have contributed to the above-average production of early acquired words. This should be investigated in future studies.

With respect to syntax, deficits in processing complex constructions have been unequivocally reported in persons with schizophrenia (Dwyer et al., 2014; Tan et al., 2016; Çokal et al., 2019; Tan and Rossell, 2019; di San et al., 2022). In addition to an association with working memory impairment, such deficits have been specifically related to positive FTD (Tan et al., 2016), but were detectable even in the absence of FTD (Dwyer et al., 2014). Considering that in healthy persons the ability to process complex sentences was associated with the maturation of the arcuate fasciculus, which is fully mature only in adulthood (Skeide et al., 2016), in persons with schizophrenia an association between reduced FA of the arcuate fasciculus and impaired syntax processing would seem plausible. However, no corresponding association has been found (Cavelti et al., 2018; meta-analysis see Cavelti et al., 2018).

In the domain of pragmatics, deficits in understanding context-dependent inferential meaning and discourse comprehension were found in the vast majority of affected individuals (Bambini et al., 2016). This seems consistent with the view that impaired use of higher-order context could lead to overreliance on semantic associations, to the detriment of interpretive understanding and ultimately impairing adaptation to complex social situations (Brown and Kuperberg, 2015, *cf.* Spitzer, 1993). Impaired metaphor explanation due to overreliance on context-unspecific, dominant word meanings was found to precede the conversion to schizophrenia (Heinz et al., 1996; Pawełczyk et al., 2021), suggesting a fundamental aspect of the disposition to psychotic experiences. Consistent with a reduced use of context based comprehension, pragmatic dysfunctions could be attributed to disturbed top-down processing (Stephane et al., 2014). Importantly, the current studies indicated close connections between inferential meaning and social cognition (Bambini et al., 2016; Fukuhara et al., 2017; Comparelli et al., 2020). The linguistically observable impairment in context use when interpreting figures of speech may represent only a partial phenomenon of a broader impairment of contextualization in psychosis. The hypothesis of reduced use of semantic context may also help to explain significantly higher rates of psychosis among migrants, refugees, and persons with minority status (Brandt et al., 2019; Henssler et al., 2020; Selten et al., 2020; Varchmin et al., 2021). Here, unknown information regarding not only semantic but also pragmatic cultural contexts may increase prediction errors and facilitate stress-associated alterations that contribute to psychotic experiences, particularly when exposed to social exclusion, discrimination and racism (Baker et al., 2021; Lazaridou et al., 2023).

Regarding pragmatic language production, current studies mainly indicate impaired use of referential coherence (Elvevåg et al., 2007; Perlini et al., 2012; Colle et al., 2013; Tang et al., 2021; Voppel et al., 2021) and cohesion (Çokal et al., 2018; Sevilla et al., 2018; García-Mieres et al., 2020; Mackinley et al., 2021). This could be due to a disrupted feedback during language production, which is expected to result in less contextual constraint and loosening of associations when disintegrated associations are incorporated into an utterance (Brown and Kuperberg, 2015; Corcoran et al., 2020). Again, experiences of linguistic prowess, cultural familiarity and stress-associated impairments need to be studied in more detail.

Apart from the language dimension, the cognitive dimension is also expected to contribute to FTD (Hart and Lewine, 2017). In fact, both positive and negative FTD have been associated with impaired attention (Nagels et al., 2016; Ucok et al., 2021; Oeztuerk et al., 2022) and problem-solving ability (Little et al., 2019; Comparelli et al., 2020). This could possibly be due to hallucinations or anxiety and should be investigated in future studies. It should be noted, that in persons with schizophrenia, particularly verbal memory functions may be affected by progressive impairments, possibly due to a reduction in gray matter in the temporal lobe, although other cognitive symptoms have been described as tendentially stable or improving over the life course (Heilbronner et al., 2016). Overall, the present results point to the need to control for cognitive performance and schooling in similar studies (*cf.* Docherty et al., 2013; Morgan et al., 2021; Kircher et al., 2022).

It seems furthermore important, that both negative and positive FTD have been associated with social sequalae (Bowie et al., 2011; Muralidharan et al., 2018; Comparelli et al., 2020; Marggraf et al., 2020; Oeztuerk et al., 2021). With respect to directionality, it is plausible that FTD cause communication impairments that lead to impairments in social functioning. Alternatively, it also seems conceivable that in the course of the disease, impaired communication, impaired social functioning and social exclusion stress may lead to a further impoverishment of the degrees of freedom of thought in the sense of a vicious circle. This seems to be supported by findings indicating compensation for disturbed top-down processes through language alignment (Sharpe et al., 2022) and improved semantic cohesion (Saavedra, 2010) during social communication. In addition, persons with schizophrenia tend to use particularly few words expressing positive emotions (Sarzynska-Wawer et al., 2021). Under socioemotional stress, they may experience a general intensification of FTD (Cohen et al., 2014), an increase in positive FTD (Minor et al., 2016), or show a decrease in information reference (Rubino et al., 2011). To elucidate whether this might be related to negative experiences, it seems prudent to control for depressive and trauma-related symptoms (*cf.* Varchmin et al., 2021) in future studies. Social stress exposure and the associated “affective reactivity” (Docherty et al., 1994) may also be related to volume reductions that particularly affect regions relevant to language (i.e., left periinsular region) and emotional processes (i.e., amygdala/medial temporal lobe cluster) (Nickl-Jockschat et al., 2011). Indeed, an association was observed between positive FTD and atrophy in areas involved in emotional processes, including the amygdala (e.g., Spalletta et al., 2010). Thus, assuming a disease (and potentially also antipsychotic-treatment-related; Ho et al., 2011; Fusar-Poli et al., 2013; Heilbronner et al., 2016) vulnerability of the associated brain regions, the language system might be particularly sensitive to emotional stress.

Speech graph measures were used in several studies to quantify speech connectivity. This method showed high discriminatory value (Mota et al., 2012; Palaniyappan et al., 2019, but *cf.* Chrobak et al., 2022) with reduced speech graph connectivity in individuals with schizophrenia compared to individuals with bipolar disorder. This has been interpreted as an indicator of disorganized speech (Mota et al., 2012; Palaniyappan et al., 2019) and may be related to negative symptoms (Mota et al., 2017; Morgan et al., 2021; Spencer et al., 2021, but *cf.* Palaniyappan et al., 2019). Reduced speech graph connectivity may also predict transition from clinical high risk to first psychotic episode (Morgan et al., 2021; Spencer et al., 2021) and to schizophrenia (Mota et al., 2017). On a critical note, the applicability seems to depend on the analyzed speech material, so that both the relation to clinically relevant speech samples and the generalizability seem somewhat limited. Also, further analyses of correlation with clinical findings would be desirable. In addition to reduced connectivity, reduced semantic content as measured by the *ELMo* or *Vector Unpacking* has been shown to be highly specific for schizophrenia (Sarzynska-Wawer et al., 2021) and to have a high predictive value (Rezaii et al., 2019). This again seems to support the view that negative FTD form a core component of schizophrenia.

Although not the focus of this review, given their overlap with language, neuromotor, affective, cognitive, and psychosocial functions, recent speech-and voice-related findings in individuals with schizophrenia will be briefly discussed: Against the background of meta-analytically demonstrable large heterogeneity of study results and strong effects of task condition (Parola et al., 2020), reduced pitch variability and increased utterance duration have been identified as the only cross-linguistically generalizable acoustic features (while longer pause duration and reduced speech rates were replicated in a subpopulation) (Parola et al., 2023). An association with negative symptoms seems of interest here, since reduced pitch variability has been related to monotone speech and interpreted as flat affect, prolonged pause duration to alogia or affective flattening and increased utterance duration to low energy and high vocal effort (Parola et al., 2023). Especially machine learning approaches reported high accuracy in discriminating between individuals with and without schizophrenia (for a review see Parola et al., 2020). In this context, next to longer pause durations (de Boer et al., 2023), persons with schizophrenia spectrum disorder exhibited a more fragmented (indicated by more voiced segments per second; de Boer et al., 2023; Voppel et al., 2023) and more monotonous (indicated by reduced spectral flux variation; de Boer et al., 2023; resp. lower frequency and volume entropy; Tahir et al., 2019) speech, as well as a more strained voice (indicated by reduced mean spectral slope of voiced and unvoiced regions; de Boer et al., 2023). Importantly, the combination of acoustic and lexico-semantic measures indicated the co-occurrence of fragmented speech and decreased semantic connectedness as indicative of schizophrenia spectrum disorder (Voppel et al., 2023).

The present findings focus largely on the deficit aspects of altered language. Given the findings of an above-average proportion of artistic professions among persons with psychotic disorders (Juda, 1949, but *cf.* Kyaga et al., 2013) or their relatives (Kyaga et al., 2011, 2013), it seems worthwhile to examine creative aspects of altered language. In particular, an overrepresentation of persons with schizophrenia or bipolar disorder among professional writers could be an indicator of beneficial effects of the loosening of traditional context restraints on linguistic creativity (Kyaga et al., 2013). Further research could illuminate this perspective and possible implications for linguistic creativity in therapeutic contexts.

## 4 Limitations

The current work has several limitations. Most importantly, the search was performed only in PubMed. Studies that are exclusively accessible via other search engines were therefore not considered. Also, studies examining children/adolescents diagnosed with schizophrenia/psychosis were not included, nor were studies primarily focusing on neuroimaging or genetics. This was done to reduce the large number of relevant studies to those with the strongest relationship to clinical work in adult psychiatry. Similarly, by focusing on clinical findings, the biolinguistic models were not presented in great detail, and no new model was derived from the data. Also, potential interventions were not addressed here, but could be of clinical importance and should therefore be included in future studies. Finally, the presentation of the results led to a deficit-oriented view of the affected individuals. For future studies and reviews, it seems advisable to balance the view by also including strengths and potentials of those affected.

## 5 Conclusion

Alterations at all levels of the language system appear to be inherent in schizophrenia and are evident both clinically as FDT and in specific linguistic analyses. Corresponding schizophrenia-specific findings reviewed here include poverty of speech with reduced word production (e.g., Cuesta and Peralta, 2011) as well as incomplete (Tang et al., 2021) and content-poor words (Sarzynska-Wawer et al., 2021), short and incomplete sentences (Perlini et al., 2012) with overall reduced expressiveness and complexity (Hong et al., 2015). Furthermore, the processing of complex syntax (e.g., Dwyer et al., 2014) as well as pragmatic language comprehension (e.g., Bambini et al., 2020) and production (e.g., Colle et al., 2013) are typically reduced. A literal understanding of a context-specific inferred understanding of unexpected proverbs and metaphors in explanation tasks can point to a general reduction of the impact of context and point to imprecise priors including alterations in semantic memory (Adams et al., 2016; Heinz et al., 2019).

Notably, already in the at-risk state, linguistic phenomena typical of schizophrenia [including disorganization (Demjaha et al., 2012), poverty of content, reduced referential cohesion (Bearden et al., 2011), difficulties in pragmatic language comprehension (Pawełczyk et al., 2019) and reduced semantic coherence (Bilgrami et al., 2022)] can be detected and appear to be of predictive value for conversion to psychosis. Thus, for clinical work, systematic language evaluation can support early detection, differential diagnosis and assessment of the degree of overall impairment. Here, speech analyses based on natural language processing are gaining importance in order to increase objectivity and precision in differential diagnosis (e.g., Mota et al., 2012) and early detection (e.g., Bilgrami et al., 2022). Of particular interest are schizophrenia-typical reduced speech graph connectivity (e.g., Morgan et al., 2021), reduced semantic density, and increased reference to auditory sensations (Rezaii et al., 2019) that may precede the development of psychosis. So far, these methods have been used in scientific studies, but not in clinical practice, and the relationship of the individual parameters to clinical findings appears to be unclear at present. Future studies should also address how to use specific information regarding linguistic alterations for prevention and therapy and how to use creativity in language use when coping with psychotic experiences.

## Author contributions

FE: Conceptualization, Data curation, Formal analysis, Investigation, Methodology, Project administration, Writing – original draft, Writing – review & editing. CM: Supervision, Validation, Writing – review & editing. KL: Supervision, Validation, Writing – review & editing. AH: Conceptualization, Project administration, Supervision, Validation, Writing – review & editing.

## Glossary

BERT: Bidirectional Encoder Representations from Transformers
*CDI*: Communication Disturbances Index
*ELMo*: Embeddings from Language Models
FTD: Formal thought disorder
*FA*: Fractional anisotropy
*K-FTDS*: Kiddie Formal Thought Disorder Rating Scale
*LCC*: Largest connected component
*LCCr*: Largest connected component (ratio between original and random connectedness)
*LSC*: Largest strongly connected component
*LSCr*: Largest strongly connected component (ratio between original and random connectedness)
*LSA*: Latent Semantic Analysis
*NLP*: Natural language processing
*PANSS*: Positive and Negative Syndrome Scale
*SANS*: Scale for the Assessment of Negative Symptoms
*SAPS*: Scale for the Assessment of Positive Symptoms
*SOA*: Stimulus Onset Asynchrony
*SIPS*: Structured Interview for Psychosis-risk Syndromes
*TALD*: Thought and Language Disorder scale
*TLI*: Thought and Language Index
*TLC*: Thought, Language, and Communication
*VF*: Verbal fluency
